# Measuring coverage and quality of supportive care for inpatient neonatal infections: EN-BIRTH multi-country validation study

**DOI:** 10.7189/jogh.12.04029

**Published:** 2022-04-30

**Authors:** Aniqa T Hossain, Shafiqul Ameen, Nahya Salim, KC Ashish, Harriet Ruysen, Tazeen Tahsina, Anisuddin Ahmed, Md Hafizur Rahman, Shema Mhajabin, Sabrina Jabeen, Kimberly Peven, Stefanie Kong, Louise T Day, Yasir B Nisar, Evelyne Assenga, Shamim A Qazi, Qazi S-u Rahman, Shams El Arifeen, Ahmed E Rahman, Joy E Lawn, EN-BIRTH Study Group

**Affiliations:** 1 International Centre for Diarrhoeal Disease Research, Bangladesh, Dhaka, Bangladesh; 2 Muhimbili University of Health and Allied Sciences (MUHAS), Muhimbili, Tanzania; 3 International Maternal and Child Health, Department of Women’s and Children’s Health, Uppsala University, Uppsala, Sweden; 4 London School of Hygiene & Tropical Medicine, London, UK; 5 World Health Organization, Geneva, Switzerland‎

## Abstract

**Background:**

An estimated 7 million episodes of severe newborn infections occur annually worldwide, with half a million newborn deaths, most occurring in low- and middle-income countries. Whilst injectable antibiotics are necessary to treat the infection, supportive care is also crucial in ending preventable mortality and morbidity. This study uses multi-country data to assess gaps in coverage, quality, and documentation of supportive care, considering implications for measurement.

**Methods:**

The EN-BIRTH study was conducted in five hospitals in Bangladesh, Nepal, and Tanzania (July 2017-July 2018). Newborns with an admission diagnosis of clinically-defined infection (sepsis, meningitis, and/or pneumonia) were included. Researchers extracted data from inpatient case notes and interviews with women (usually the mothers) as the primary family caretakers after discharge. The interviews were conducted using a structured survey questionnaire. We used descriptive statistics to report coverage of newborn supportive care components such as oxygen use, phototherapy, and appropriate feeding, and we assessed the validity of measurement through survey-reports using a random-effects model to generate pooled estimates. In this study, key supportive care components were assessment and correction of hypoxaemia, hyperbilirubinemia, and hypoglycaemia.

**Results:**

Among 1015 neonates who met the inclusion criteria, 89% had an admission clinical diagnosis of sepsis. Major gaps in documentation and care practices related to supportive care varied substantially across the participating hospitals. The pooled sensitivity was low for the survey-reported oxygen use (47%; 95% confidence interval (CI) = 30%-64%) and moderate for phototherapy (60%; 95% CI = 44%-75%). The pooled specificity was high for both the survey-reported oxygen use (85%; 95% CI = 80%-89%) and phototherapy (91%; 95% CI = 82%-97%).

**Conclusions:**

The women's reports during the exit survey consistently underestimated the coverage of supportive care components for managing infection. We have observed high variability in the inpatient documents across facilities. A standardised ward register for inpatient small and sick newborn care may capture selected supportive care data. However, tracking the detailed care will require standardised individual-level data sets linked to newborn case notes. We recommend investments in assessing the implementation aspects of a standardised inpatient register in resource-poor settings.

An estimated 7 million episodes of newborn (possibly severe) infections occur globally each year, including sepsis, pneumonia, and meningitis [1]. Most newborn infection deaths occur in low- and middle-income countries (LMICs) and are preventable [2], resulting in approximately half a million neonatal deaths [3-5]. Appropriate and timely treatment with antibiotics is vital for neonatal infection management, along with high-quality supportive care, so that even the sickest newborns can survive and thrive [6]. Common challenges these newborns face include hypothermia, hypoxaemia (low oxygen saturation), hyperbilirubinemia (jaundice), and hypoglycaemia. Hypoxaemia, or low oxygen saturation (SpO_2_<90%), is common among newborns with infections, neonatal encephalopathy, and preterm [7,8]. Hypoxaemia has wide variations in prevalence across different clinical severity classifications and geographical areas and is one of the strongest predictors of neonatal mortality, with a six times higher likelihood of death [9,10]. Most hypoxaemia-related deaths can be averted by routine assessment, timely detection, and early correction through appropriate oxygen therapy or respiratory support [11,12]. Jaundice identification and treatment in LMICs for newborns with sepsis can prevent serious complications [13,14]. Serum bilirubin should be checked for all newborns admitted to the hospital with a serious bacterial infection. Severe hyperbilirubinemia can be treated with phototherapy [15,16]. Neonatal hypoglycaemia, defined as a blood glucose level of <40 mg/dL [17], requires early detection to prevent complications, especially with concurrent risk factors of prematurity, intrauterine growth restriction, and maternal diabetes. Supporting breastfeeding and other assisted newborn feeding may be an initial treatment for hypoglycaemia [18].

Supportive care components are described in the World Health Organisation (WHO) Standards for improving the quality of care for small and sick newborns in health facilities at different levels of health systems (**Figure 1**), and several indicators are assessed in this study, notably for safe oxygen therapy, phototherapy for jaundice (if needed), and management of hypoglycaemia. [19-22].

**Figure 1:**
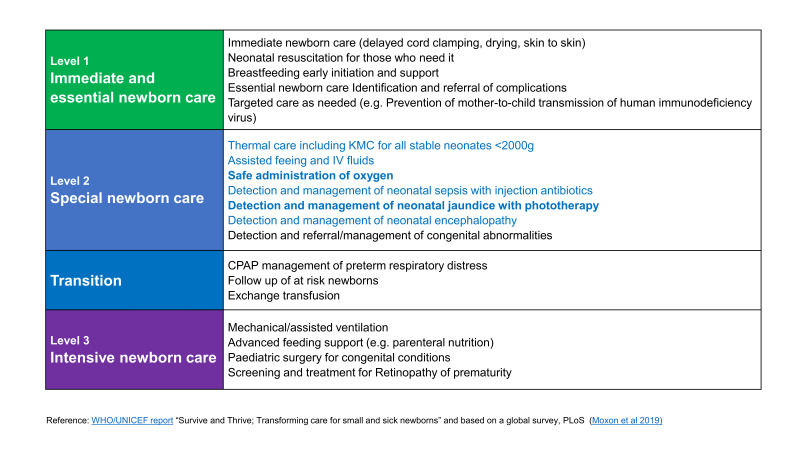
World Health Organisation (WHO) Levels of Newborn Care with Interventions.

Reliable data are vital for individual clinical management of newborns with infection and improve the quality and coverage of care. Such data are essential for tracking overall progress towards the SDGs, the Every Newborn Action Plan (ENAP) [23], and other commitments [24] to end preventable newborn deaths. LMICs have relied on population-based surveys [25,26] to track health outcomes and contact coverage. However, there are concerns regarding the accuracy of recall-based estimation for intervention coverage, especially for more complex clinical practices, including infection management [3,27-29]. Population-based surveys such as the Demographic and Health Surveys (DHS) Program and Multiple Indicator Cluster Surveys (MICS) provide data on coverage for health care use, including data on management of childhood illnesses in low-resource countries [30,31]. Studies have revealed low accuracy for population-based survey reports of caregiver-reported symptoms of childhood illnesses, especially among newborns [27,28]. Given the high proportion of care now in hospitals, routine health management information systems (HMIS) can track coverage of key interventions, including supportive care, yet there are concerns including irregular and incomplete documentation and reporting practices. We have previously reported on the validation of survey-reports for antibiotic use for inpatient neonatal infection management [3,32]. But, as yet, there is little evidence regarding the capturing of infection management practices in LMICs through the routine health information system, with irregular and incomplete documentation and reporting practices.

## METHODS

The Every Newborn Birth Indicators Research Tracking in Hospitals (EN-BIRTH) study was an observational study conducted between July 2017 and July 2018 in five referral hospitals: Maternal and Child Health Training Institute (MCHTI), Azimpur and Kushtia General Hospital in Bangladesh (BD), Pokhara Academy Health Sciences in Nepal (NP), and Muhimbili National Hospital and Temeke District Hospital in Tanzania (TZ). The EN-BIRTH study's objectives, design, data collection, and results are described elsewhere [3,33].

### Study population and inclusion criteria

Admitted newborns aged ≤28 days at admission, weighing >1500g at admission or discharge, or with a gestational age of >32 weeks, receiving management for clinically-defined infections (sepsis, pneumonia, meningitis) documented in the individual case notes were included in the study. Major congenital abnormality or neonatal encephalopathy (“severe asphyxia”) were considered as exclusion criteria. All women were interviewed as primary caregivers (mainly mothers) at exit from the hospital after their newborns were discharged.

### Data collection

Clinically trained data collectors extracted data from hospital inpatient case notes using a tablet-based structured checklist. Separately trained data collectors interviewed caretakers of sick newborns with a structured questionnaire. Data were collected using a custom-built electronic data capture system. [34]. To determine the reliability of our data, Cohen’s Kappa coefficients of agreement were calculated for a 5% subset cases to study supervisors interviewed/extracted data for comparison with the data collector’s findings.

### Ethical consideration

Ethical approval was obtained from the institutional review boards in all operating countries, in addition to the London School of Hygiene & Tropical Medicine (Table S1 In the **Online Supplementary Document**). Voluntary informed and written consent was obtained from caretakers before exit interviews. Confidentiality and anonymity were maintained at each stage of data management and analysis.

### Data analyses

We used a Structured Query Language (SQL) server for data storage and management and the Stata statistical software package (version 14) for data analysis [35]. STROBE checklists for observational studies [36] were used to report enrolment flow (Table S2 in the **Online Supplementary Document**). We reported the background characteristics of newborns and their caregivers through descriptive statistics. Asset scores were generated using the standard Principal Component Analysis procedure [37]. The EN-BIRTH larger data set was used for country-specific assignment of wealth quintile to the neonatal infection cases.

We focused on measures to diagnose and track supportive treatment specifically for hypoxaemia, hyperbilirubinemia, and hypoglycaemia as the critical components of supportive care. We calculated the point prevalence of intervention coverage with 95% confidence intervals (CI) based on the hospital inpatient case notes. We reported estimates separately for each of the five facilities and a pooled estimate using a random-effects model.

We compared the women’s exit-survey report with the extracted data from inpatient case note verification as the “Gold Standard”. For validity of measurements, sensitivity, specificity, and percent agreement were reported with a 95% CI for key supportive care components by each facility. The percentage of women answering “don’t know” to survey questions was calculated and analysed, with “don’t know” considered as “no”. Sensitivity and specificity analyses were performed if the column total counts in the two-way tables were more than 10.

## RESULTS

**Figure 2:**
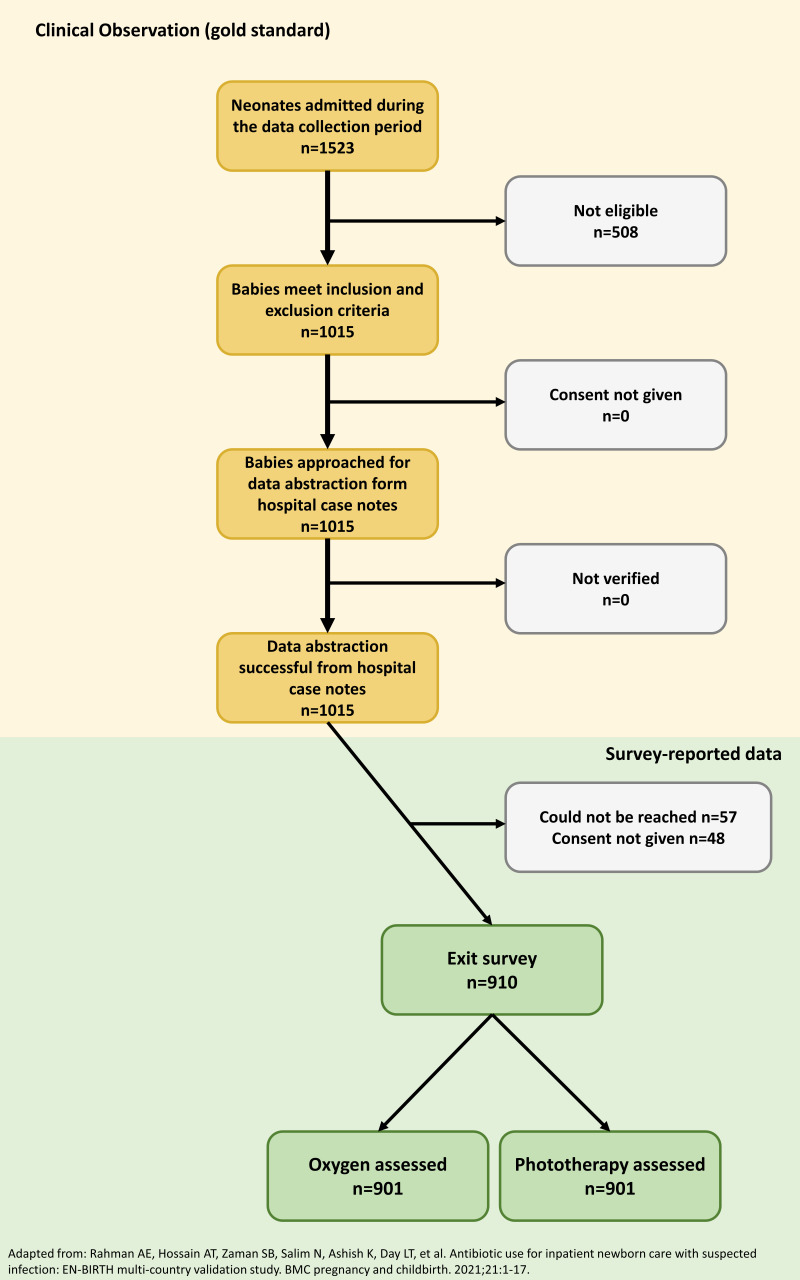
EN-BIRTH study flow diagram for newborns treated with severe infections (n = 1015).

Case note data were abstracted for the 1015 neonates meeting the inclusion criteria (n = 409 from BD, n = 344 from NP, n = 262 from TZ). Primary caretakers of all eligible neonates were approached, and 90% (n = 910) completed an exit interview survey (**Figure 2**). Of those who did not complete the exit interview survey, 5% (n = 57) were not reached by data collectors, and 5% (n = 48) did not provide consent. In addition, we approached all caretakers of the neonates and successfully interviewed 910 (90%) of them before discharge. Unfortunately, we could not reach 57 (5%) women, and 48 (5%) women did not consent to participate in the study. **Figure 2** summarises the flow of the selection process of the overall sample in this analysis.

**Table 1** presents the neonate’s background characteristics, clinical history, and newborn physical examination as recorded in the hospital inpatient case notes. The most common clinical infection diagnosis was sepsis (Azimpur, BD = 78%; Kushtia, BD = 77%; Pokhara, NP and Temeke, TZ = 99%; Muhimbili, TZ = 76%). Around 10% of the cases in Nepal and Tanzania had jaundice on admission, along with other comorbidities. Most included newborns were <7 days old (50%-75%), except in Muhimbili TZ (25%), where 47% were between 7-13 days old. Low birth weight (<2500g) ranged from 12% in Muhimbili, TZ to 36% in Kushtia, BD. Birthweight was recorded for more than 87% of newborns. Admission weight was not recorded in the case notes for less than 10% cases in Azimpur, BD and Kushtia, BD, 22.4% in Muhimbili, TZ, and more than 70% in Pokhara, NP and Temeke, TZ.

**Table 1:** Characteristics of newborns in inpatient wards, case note verification, EN-BIRTH study (n = 1015 children)

	Bangladesh (BD)		Nepal (NP)	Tanzania (TZ)	
	**Azimpur Tertiary**	**Kushtia District**	**Pokhara Regional**	**Temeke Regional**	**Muhimbili National**
	**n = 106**	**n = 303**	**n = 344**	**n = 213**	**n = 49**
	**n (%)**	**n (%)**	**n (%)**	**n (%)**	**n (%)**
**Age**					
≤6 d	67 (63.2)	151 (49.8)	259 (75.3)	153 (71.8)	12 (24.5)
7-13 d	17 (16)	60 (19.8)	42 (12.2)	34 (16)	23 (46.9)
14-20 d	13 (12.3)	42 (13.9)	19 (5.5)	13 (6.1)	9 (18.4)
21-28 d	9 (8.5)	50 (16.5)	24 (7)	13 (6.1)	5 (10.2)
**Sex:**					
Male/Boy	59 (55.7)	183 (60.4)	225 (65.4)	127 (59.6)	30 (61.2)
**Birth weight:**					
1500-2000 g	7 (6.6)	38 (12.5)	15 (4.4)	14 (6.6)	4 (8.2)
2001-2500 g	20 (18.9)	72 (23.8)	54 (15.7)	30 (14.1)	2 (4.1)
2500+ g	65 (61.3)	165 (54.5)	263 (76.5)	165 (77.5)	40 (81.6)
Not recorded	14 (13.2)	28 (9.2)	12 (3.5)	4 (1.9)	3 (6.1)
**Clinical History:**					
Not feeding well	43 (40.6)	37 (12.2)	42 (12.2)	77 (36.2)	18 (36.7)
Lethargy/reduced consciousness	6 (5.7)	2 (0.7)	16 (4.7)	14 (6.6)	9 (18.4)
Convulsion	3 (2.8)	8 (2.6)	12 (3.5)	21 (9.9)	7 (14.3)
Fever	44 (41.5)	25 (8.3)	211 (61.3)	127 (59.6)	20 (40.8)
Respiratory distress or fast breathing	36 (34)	35 (11.6)	45 (13.1)	20 (9.4)	7 (14.3)
**Physical examination:**					
Fever (>38 degree)	28 (26.4)	298 (98.3)	172 (50)	81 (38)	10 (20.4)
Hypothermia (<35 degree)	3 (2.8)	0 (0)	0 (0)	1 (0.5)	0 (0)
Respiratory Rate (>60/min)	40 (37.7)	23 (7.6)	135 (39.2)	29 (13.6)	9 (18.4)
Bulging Fontanelle	0 (0)	0 (0)	0 (0)	2 (0.9)	2 (4.1)
Umbilical redness or draining pus	8 (7.5)	0 (0)	3 (0.9)	5 (2.3)	3 (6.1)
Skin Pustules	2 (1.9)	2 (0.7)	11 (3.2)	3 (1.4)	3 (6.1)
**Diagnosis at admission:**					
Sepsis	83 (78.3)	233 (76.9)	341 (99.1)	211 (99.1)	37 (75.5)
Pneumonia	23 (21.7)	70 (23.1)	1 (0.3)	1 (0.5)	8 (16.3)
Meningitis	0 (0)	0 (0)	2 (0.6)	1 (0.5)	4 (8.2)
**Jaundice with other comorbidities**	2 (1.9)	5 (1.7)	33 (9.6)	20 (9.4)	6 (12.2)

### Assessment and supportive care for hypoxaemia or low oxygen saturation

**Figure 3:**
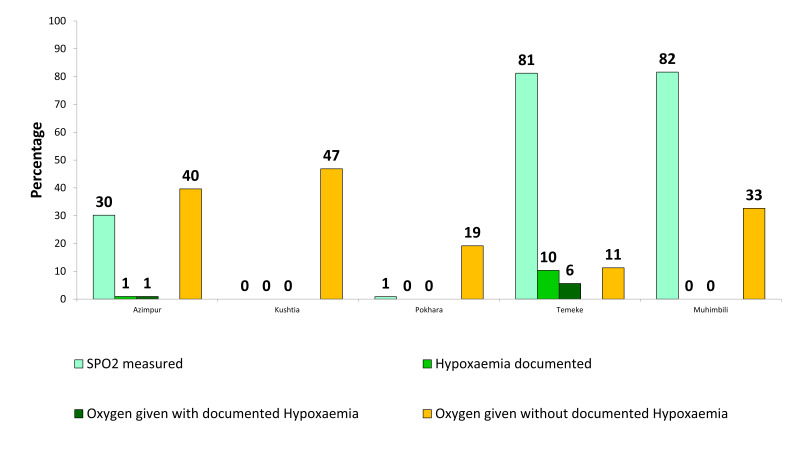
Gaps in coverage, quality of care and documentation practice related to assessment and correction of hypoxaemia (n = 1015).

**Figure 3** presents the gaps in coverage, quality of care, and documentation practice related to the assessment and correction of hypoxaemia. SpO_2_ assessment varied substantially between facilities, 81% in Temeke, 82% in Muhimbili, 30% in Azimpur, and 1% in Pokhara. Very few neonates had documented hypoxaemia in Bangladesh, Nepal, and Muhimbili. The use of oxygen therapy was high in Muhimbili, TZ, Kushtia and Azimpur, BD, where oxygen was given in the absence of a documented hypoxaemia diagnosis (40% in Azimpur, BD, 47% in Kushtia, BD, and 33% in Muhimbili, TZ).

### Assessment and treatment for jaundice

**Figure 4:**
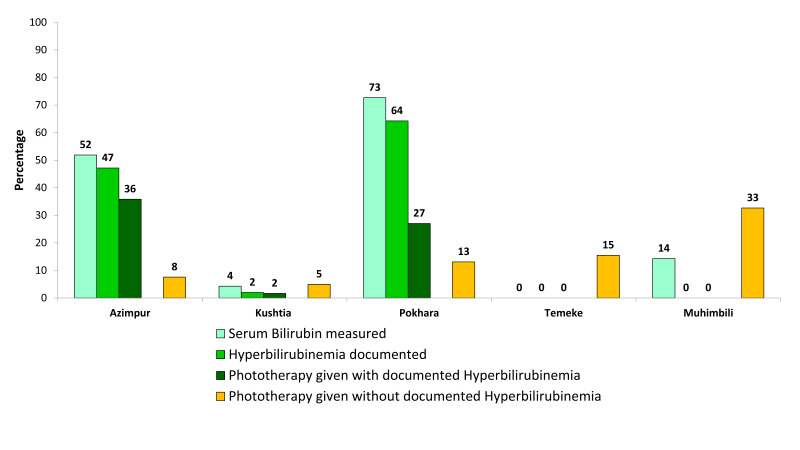
Gaps in coverage, quality of care and documentation practice related to assessment and correction of hyperbilirubinemia (n = 1015).

**Figure 4** shows the gaps in coverage, quality of care, and documentation practice related to assessment and correction of jaundice. Evaluation of jaundice varied substantially across facilities. While more than half of admitted newborns had their serum bilirubin measured in Azimpur, BD (52%) and Pokhara, NP (73%), coverage was low (<14%) in other hospitals. In Azimpur, BD, where 47% of newborns had documented hyperbilirubinemia, 36% were given phototherapy, while 8% were without documented hyperbilirubinemia. Despite 64% of newborns having documented hyperbilirubinemia in Pokhara, NP, 40% were treated with phototherapy, one-third of whom did not have documented hyperbilirubinemia. In hospitals where serum bilirubin measurement was uncommon, treatment with phototherapy ranged from 5%-33%, mostly for newborns without documented hyperbilirubinemia.

### Assessment and treatment for hypoglycaemia

**Figure 5:**
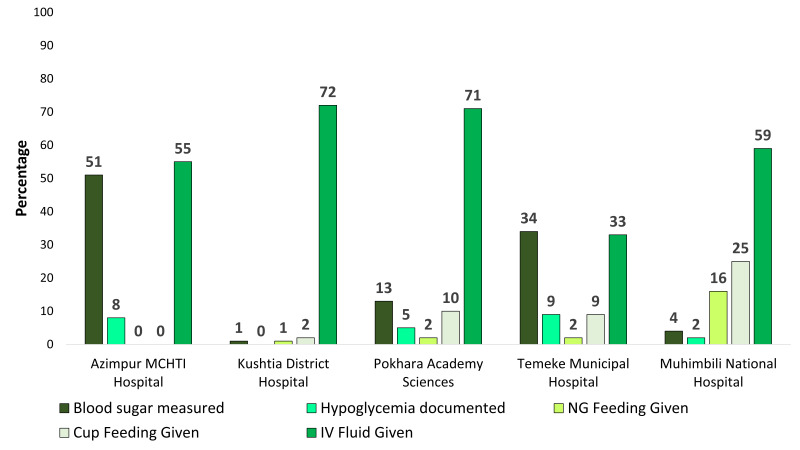
Gaps in coverage, quality of care and documentation practice related to assessment and correction of hypoglycaemia (n = 1015).

**Figure 5** presents the gaps in coverage, quality of care, and documentation practice related to assessing and treating hypoglycaemia. Blood glucose measurement ranged from less than 1% in Kushtia, BD, to 51% in Azimpur, BD. Intravenous fluid administration ranged from 33% in Temeke, TZ to 72% in Kushtia, BD.

**Table 2** shows the validation results of women’s exit-interview survey reports. Overall sensitivity was 47% (95% CI = 30%-64%) for oxygen use, and specificity was 85% (95% CI = 80%-89%). In Kushtia, BD, the sensitivity of reporting for the provision of oxygen was particularly low – 29% (95% CI = 22%-37%). An exit interview survey measuring the use of phototherapy had overall moderate sensitivity of 59% (95% CI = 44%-75%) and overall high specificity of 91% (95% CI = 82%-97%). Sensitivity was highest in Pokhara, NP – 73% (95% CI = 64%-80%), and specificity was high in Muhimbili, TZ – 96% (95% CI = 79%-99%). Thus, the level of agreement was moderate to high for both supportive care components. Table S3 in the **Online Supplementary Document** presents the detailed individual-level validation in exit survey for selected indicators. Table S4 in the **Online Supplementary Document** presents the heterogeneity statistics (I^2^ and Tau^2^ values) from pooled analysis.

**Table 2:** Individual-level validation in exit survey for supportive care to assess the accuracy of women's report

Indicator		Bangladesh		Nepal	Tanzania		All sites pooled (Random effects)
		**Azimpur MCHTI Hospital**	**Kushtia District Hospital**	**Pokhara Academy Sciences**	**Temeke Municipal Hospital**	**Muhimbili National Hospital**	**All sites**
**Oxygen given**	Sensitivity (95% CI)	52.5 (36.1, 68.5)	29.1 (21.7, 37.3)	64.4 (50.9, 76.4)	36 (18, 57.5)	58.3 (27.7, 84.8)	47.1(30.4, 64.1)
	Specificity (95% CI)	85.5 (74.2, 93.1)	81.6 (74.7, 87.3)	83.3 (78.1, 87.6)	91.5 (84.8, 95.8)	72.7 (49.8, 89.3)	84.8(79.9, 89.1)
	Agreement (%)	72.5	56.9	79.7	81.7	67.6	72.3(60.7, 82.6)
**Phototherapy given**	Sensitivity (95% CI)	58.7 (43.2, 73)	65 (40.8, 84.6)	72.7 (64.1, 80.2)	-*	-*	59.8(44.1, 74.7)
	Specificity (95% CI)	89.3 (78.1, 96)	96.8 (94, 98.5)	80.9 (74.5, 86.2)	-*	-*	90.7(82.1, 96.8)
	Agreement (%)	75.5	94.6	77.5	81.0	88.2	84.1(73.8, 92.3)

*Validation not done because not having at least ten observations in either column of the two-by-two table.

## DISCUSSION

Whilst research examining the measurement of antibiotic use for infection management had already been published [3], this is the first study to examine gaps in documentation and quality of supportive care for neonatal infections. More than 1000 admitted newborns were included in our analysis from five hospitals in Bangladesh, Nepal, and Tanzania, comparing inpatient case notes and women's reports during an exit interview surveys for validation [38]. We found that the documentation practice in the case notes regarding supportive care components was poor or sub-optimal. Documentation practices varied across different facilities and different care components. In all five facilities in the three countries, recorded supportive care practices were low. Our assessment validating women's reports on the exit survey showed low sensitivity for reported management of hypoxaemia and hyperbilirubinemia.

Women's reports from the exit survey under-reported the case notes documented coverage of supportive care interventions. Little may have been communicated to the women about their newborn infection management. If so, this lack of communication makes it difficult for the mothers to report on what is being done for them or their newborns. This was supported by EN-BIRTH findings that showed high “don’t know” responses when assessing the accuracy of exit survey measures of antibiotic treatment for neonatal infection [3]. Lack of an easily measurable denominator compounds measurement challenges for tracking interventions targeting small and sick newborns, because it is not possible to accurately capture the number of newborns in need of the interventions. Other studies also suggest that population-based research cannot accurately capture the number of neonatal infections, which is similar to our understanding from this study [39]. Moreover, it would be very expensive to use a large enough sample in nationally representative surveys, given that only a relatively small number of babies will require these specific interventions. Given the low sensitivities found for these supportive treatment measures, we do not recommend incorporating these indicators in the national surveys [30,31].

Basic diagnostics are fundamental to ensuring appropriate infection management and supportive care [40], yet there was a lack of necessary bedside diagnostics in these hospitals [41]. Diagnostic components are generally more available in the facilities located in Dhaka than in other districts in Bangladesh. For hyperbilirubinemia management, Pokhara, NP performed better than other facilities. This may have been related to some specific initiatives for strengthening newborn care in Nepal during this study.

Insufficient documentation of clinical interventions is common, even in high-income countries [42]. Hence it is plausible that some interventions were given to the neonates, but were not well documented. Our findings indicate documentation gaps in all these sites, which may mask true gaps in coverage and quality. We did not find any documented test or examination results for many of the neonates who received intervention or treatment. In contrast, some newborns with infection did not receive the required intervention (SpO_2_ assessment/phototherapy), as documented in their case notes. These interventions can be costly from both the health system and societal perspective [43]. This study observed evidence of oxygen given when no hypoxaemia was documented. This may be due to a gap in documentation (i.e., true hypoxaemia without documentation in case notes); however, if this difference was due to inappropriate treatment, this might have augmented the health care expenditure for both receiver and providers. However, based on the data available from this study, the cause of the gaps cannot be determined. Further studies are needed to assess whether these gaps result from inappropriate care or lack of proper documentation at the facility level.

The perceived importance of the documentation of care components is low or non-existent [44]. There is also a question of appropriate capacity and documentation skills of the providers, which could not be ensured in these study settings [45]. The patient load in some of these public facilities may prohibit providers from effective documentation [46]. Moreover, there is an overall reluctance in the culture of facility setting for documentation practice. Therefore, no care provider feels the importance of documenting the provision of supportive care components. Furthermore, multiple guidelines exist for outpatient care management, but there is no standard case-note structure for inpatients, and we observe an apparent void in the overall standardisation in documentation practice for infection management with supportive care. We observe a lack of resources in the public health facility setting, where patient load is very high and critical patients are often referred to these hospitals [47]. Thus, these hospital's free-of-cost provision of care is often a double-edged sword.

Hospital records can be a data source for tracking the coverage of supportive care for neonatal infection management [48]. A mixed-methods assessment in EN-BIRTH study sites found gaps in the design of hospital inpatient case notes and inconsistencies in documentation practices between facilities and by various health service providers [3,44]. A standard clinical register for admission/discharge of inpatient sick newborns may improve consistency [49] and contribute to a better quality of care [50-52]. However, such clinical registers can only include a shortlist of variables, usually entered at admission and discharge/deaths. Shifting towards standard inpatient records and adopting new technologies designed for resource-poor settings could improve documentation and reduce the workload for documentation [53]. However, setting up and managing an advanced database can be challenging and requires adequate resourcing and care regarding data governance and confidentiality [54,55].

### Strengths and Limitations

The strengths of our study are the large sample size, the multi-country sites, and the focus on coverage measurement for a high-impact intervention. Data collection was conducted by trained researchers using a tablet to optimise data management. Therefore, along with the sensitivity specificity, we have also reported the percent agreement in order to have one uniform measure of agreement across all indicators and sites. It is also important to acknowledge that case note documentation has limitations in both high- and low-resource settings [56]. Therefore, the validity assessment may have been influenced by the possible imprecision of case note documentation. While the case notes may have documentation gaps regarding what treatments were provided, it is unlikely they over-report the coverage. Therefore, the true coverages may be higher than what has been reported. This analysis cannot confirm that the identified gaps are caused by the poor documentation quality or the lack of coverage of supportive care interventions. Furthermore, indicators for corrective measures of hypoglycaemia such as nasogastric feeding, intravenous feeding, or cup feeding did not have enough observations in each column of the two-way table to report individual-level validity statistics in our paper.

## CONCLUSIONS

Appropriate quality of supportive care is indispensable for newborns with infection to prevent mortality and reduce long-term morbidity. However, adequate and structured documentation through the inpatient register is necessary to understand the gaps and deficiencies in the quality of care, informing the health care providers and policymakers. Implementation research with economic evaluation of a parsimonious data system in a range of contexts will be key to transforming data systems for the health of every newborn everywhere.

## Additional material


Online Supplementary Document
