# Supplementary material for: Measuring coverage and quality of supportive care for inpatient neonatal infections: EN-BIRTH multi-country validation study
Source: J Glob Health. 2022 Apr 30;12:04029. doi: 10.7189/jogh.12.04029 (PMC9079780; doi:10.7189/jogh.12.04029)
Supplement: Online Supplementary Document [file jogh-12-04029-s001.pdf]

Supplementary Materials

Table S1: Ethical approval of local institutional review boards, EN-BIRTH study

Teams by Country	Institutional Review Boards	Date	Number/Ref
UK	London School of Hygiene & Tropical Medicine (LSHTM) Interventions Research Ethics Committee	03/10/16	11780
Bangladesh	Icddr,b Research review Committee<br>Icddr,b ethical review committee	11/08/16<br>14/11/16	PR 16055
Nepal	Nepal Health Research Council (NHRC)	08/08/16	187/2016
Tanzania	National Institute for Medical Research (NIMRI)	20/01/17	NIMR/HQ/R.8a/Vol IX/2394
	Ifakara Health Institute	20/10/16	IHI/IRB/No: 032-2016
	Muhimbili University of Health and Allied Sciences research and Publications committee	21/10/16	2016-10-21-/AEC/Vol.XI/310

Voluntary informed consent was obtained from all participants and their care providers. All women were provided with a description of the study procedures in their preferred language at admission, and offered the right to refuse, or withdraw consent at any time during the study. Facility staff were identified before data collection began and approached for recruitment and consent. No health worker refused participation and all maintained the right to withdraw throughout the study.

This study was granted ethical approval by institutional review boards in all operating counties in addition to the London School of Hygiene & Tropical Medicine.

Table S2: STROBE Statement—Checklist of items that should be included in reports of observational studies

	Item No	Recommendation	Achieved
**Title and abstract**	1	(*a*) Indicate the study's design with a commonly used term in the title or the abstract	Yes
		(*b*) Provide in the abstract an informative and balanced summary of what was done and what was found	Yes
**Introduction**			
Background/rationale	2	Explain the scientific background and rationale for the investigation being reported	Yes
Objectives	3	State specific objectives, including any pre-specified hypotheses	Yes
**Methods**			
Study design	4	Present key elements of study design early in the paper	Yes
Setting	5	Describe the setting, locations, and relevant dates, including periods of recruitment, exposure, follow-up, and data collection	Yes
Participants	6	(*a*) *Cohort study*—Give the eligibility criteria, and the sources and methods of selection of participants. Describe methods of follow-up	Yes

		Case-control study—Give the eligibility criteria, and the sources and methods of case ascertainment and control selection. Give the rationale for the choice of cases and controls<br>*Cross-sectional study*—Give the eligibility criteria, and the sources and methods of selection of participants	
		(*b*) *Cohort study*—For matched studies, give matching criteria and number of exposed and unexposed<br>*Case-control study*—For matched studies, give matching criteria and the number of controls per case	NA
Variables	7	Clearly define all outcomes, exposures, predictors, potential confounders, and effect modifiers. Give diagnostic criteria, if applicable	Yes
Data sources/ measurement	8*	For each variable of interest, give sources of data and details of methods of assessment (measurement). Describe comparability of assessment methods if there is more than one group	Yes
Bias	9	Describe any efforts to address potential sources of bias	Yes
Study size	10	Explain how the study size was arrived at	In Protocol paper
Quantitative variables	11	Explain how quantitative variables were handled in the analyses. If applicable, describe which groupings were chosen and why	Yes
Statistical methods	12	(*a*) Describe all statistical methods, including those used to control for confounding	Yes
		(*b*) Describe any methods used to examine subgroups and interactions	Yes
		(*c*) Explain how missing data were addressed	NA
		(*d*) *Cohort study*—If applicable, explain how loss to follow-up was addressed<br>*Case-control study*—If applicable, explain how matching of cases and controls was addressed<br>*Cross-sectional study*—If applicable, describe analytical methods taking account of sampling strategy	NA
		(*e*) Describe any sensitivity analyses	Yes

**Results**			
Participants	13*	(a) Report numbers of individuals at each stage of study—eg numbers potentially eligible, examined for eligibility, confirmed eligible, included in the study, completing follow-up, and analysed	Yes
		(b) Give reasons for non-participation at each stage	Yes
		(c) Consider use of a flow diagram	Yes
Descriptive data	14*	(a) Give characteristics of study participants (eg demographic, clinical, social) and information on exposures and potential confounders	Yes
		(b) Indicate number of participants with missing data for each variable of interest	Yes
		(c) *Cohort study*—Summarise follow-up time (eg, average and total amount)	NA
Outcome data	15*	*Cohort study*—Report numbers of outcome events or summary measures over time	NA
		Case-control study—Report numbers in each exposure category, or summary measures of exposure	NA
		Cross-sectional study—Report numbers of outcome events or summary measures	NA
Main results	16	(*a*) Give unadjusted estimates and, if applicable, confounder-adjusted estimates and their precision (eg, 95% confidence interval). Make clear which confounders were adjusted for and why they were included	Yes

		(*b*) Report category boundaries when continuous variables were categorized	Yes
		(*c*) If relevant, consider translating estimates of relative risk into absolute risk for a meaningful time period	NA
Other analyses	17	Report other analyses done—eg analyses of subgroups and interactions, and sensitivity analyses	Yes
**Discussion**			
Key results	18	Summarise key results with reference to study objectives	Yes
Limitations	19	Discuss limitations of the study, taking into account sources of potential bias or imprecision. Discuss both direction and magnitude of any potential bias	Yes
Interpretation	20	Give a cautious overall interpretation of results considering objectives, limitations, multiplicity of analyses, results from similar studies, and other relevant evidence	Yes
Generalisability	21	Discuss the generalisability (external validity) of the study results	Yes
**Other information**			
Funding	22	Give the source of funding and the role of the funders for the present study and, if applicable, for the original study on which the present article is based	Yes

*Give information separately for cases and controls in case-control studies and, if applicable, for exposed and unexposed groups in cohort and cross-sectional studies.

Note: An Explanation and Elaboration article discusses each checklist item and gives methodological background and published examples of transparent reporting. The STROBE checklist is best used in conjunction with this article (freely available on the Web sites of PLoS Medicine at http://www.plosmedicine.org/, Annals of Internal Medicine at http://www.annals.org/, and Epidemiology at http://www.epidem.com/). Information on the STROBE Initiative is available at www.strobe-statement.org.

Table S3: Individual-level validation in exit survey for selected indicators, EN-BIRTH study

**Indicator**		**Bangladesh**		**Nepal**	**Tanzania**		**All sites pooled (Random effects)**
		**Azimpur MCHTI Hospital**	**Kushtia District Hospital**	**Pokhara Academy Sciences**	**Temeke Municipal Hospital**	**Muhimbili National Hospital**	**All sites**
**Oxygen given**	Sensitivity (95% CI)	52.5 (36.1,68.5)	29.1 (21.7,37.3)	64.4 (50.9,76.4)	36 (18,57.5)	58.3 (27.7,84.8)	47.1(30.4,64.1)
	Specificity (95% CI)	85.5 (74.2,93.1)	81.6 (74.7,87.3)	83.3 (78.1,87.6)	91.5 (84.8,95.8)	72.7 (49.8,89.3)	84.8(79.9,89.1)
	Agreement (%)	72.5	56.9	79.7	81.7	67.6	72.3(60.7,82.6)
**Any diagnostic test done**	Sensitivity (95% CI)	-[2]	48 (27.8,68.7)	-[2]	-[1]	-[1]	69(40.3,91.7)
	Specificity (95% CI)	-[2]	74.6 (69,79.7)	-[2]	-[1]	-[1]	53.3(30.2,75.7)
	Agreement (%)	79.4	72.4	89.7	51.4	73.5	74.4(59.3,87)
	Sensitivity (95% CI)	-[2]	-[2]	-[2]	-[2]	-[1]	8.2(0,29.2)

Indicator		Bangladesh		Nepal	Tanzania		All sites pooled (Random effects)
		Azimpur MCHTI Hospital	Kushtia District Hospital	Pokhara Academy Sciences	Temeke Municipal Hospital	Muhimbili National Hospital	All sites
**Diagnostic test done- Blood test**	Specificity (95% CI)	-[2]	-[2]	-[2]	-[2]	-[1]	97.7(93,100)
	Agreement (%)	58.8	94.8	87.5	62.1	94.1	83.9(60.2,98.2)
**Feeding support- NG feed**	Sensitivity (95% CI)	-[2]	-[2]	-[2]	-[2]	-[2]	44.3(11.7,79.1)
	Specificity (95% CI)	-[2]	-[2]	-[2]	-[2]	-[2]	98.3(96.9,99.4)
	Agreement (%)	100.0	96.7	97.2	97.2	82.4	97.1(93.9,99.2)
**Feeding support- IV fluid**	Sensitivity (95% CI)	-[2]	33.2 (27,39.9)	-[2]	-[2]	-[2]	15.5(3.3,33.9)
	Specificity (95% CI)	-[2]	81.7 (71.6,89.4)	-[2]	-[2]	-[2]	93(85.5,98.1)
	Agreement (%)	61.8	46.5	31.3	67.6	38.2	49.2(35,63.6)
**Feeding support- Cup feeding**	Sensitivity (95% CI)	-[2]	-[2]	22.6 (9.6,41.1)	-[2]	-[2]	38.4(15.3,64.2)
	Specificity (95% CI)	-[2]	-[2]	88.8 (84.5,92.2)	-[2]	-[2]	83.9(69.7,94.3)
	Agreement (%)	98.0	69.6	82.3	84.5	58.8	81.4(68.9,91.3)
**Phototherapy given**	Sensitivity (95% CI)	58.7 (43.2,73)	65 (40.8,84.6)	72.7 (64.1,80.2)	-[2]	-[2]	59.8(44.1,74.7)
	Specificity (95% CI)	89.3 (78.1,96)	96.8 (94,98.5)	80.9 (74.5,86.2)	-[2]	-[2]	90.7(82.1,96.8)
	Agreement (%)	75.5	94.6	77.5	81.0	88.2	84.1(73.8,92.3)

1= validation not done because Don't Know response more than 20%

2= validation not done because not having at least ten observations in either column of the two-by-two table

Table S4: Heterogeneity statistics for from pooled analysis

Indicator		Pooled estimate	Heterogeneity statistic	df	p-value	i2	tau2
**Oxygen given**	Gold standard coverage	30.9(18,45.4)	84.3	4	0.00	95.3	0.1
	Survey reported coverage	24.5(18.2,31.4)	18.3	4	0.00	78.2	0.0
	Don’t Know response in survey	4.6(2.4,7.5)	10.6	4	0.03	62.1	0.0
	Agreement	72.3(60.7,82.6)	48.1	4	0.00	91.7	0.1
	Sensitivity	47.1(30.4,64.1)	25.4	4	0.00	84.3	0.1
	Specificity	84.8(79.9,89.1)	8.4	4	0.08	52.5	0.0
	Positive Predictive Value	55.1(46.4,63.7)	5.6	4	0.23	28.4	0.0
	Negative Predictive Value	78.1(60.5,91.7)	88.8	4	0.00	95.5	0.2

**Any diagnostic test done**	Gold standard coverage	61.8(19.2,95.6)	760.8	4	0.00	99.5	1.0
	Survey reported coverage	61(25.8,90.7)	438.2	4	0.00	99.1	0.7
	Don't Know response in survey	13.4(2.5,30.4)	142.3	4	0.00	97.2	0.2
	Agreement	74.4(59.3,87)	82.4	4	0.00	95.1	0.1
	Sensitivity	69(40.3,91.7)	120.3	4	0.00	96.7	0.4
	Specificity	53.3(30.2,75.7)	45.8	4	0.00	91.3	0.2
	Positive Predictive Value	68.6(29.2,96.9)	270.6	4	0.00	98.5	0.8
	Negative Predictive Value	49.2(15.3,83.5)	132.4	4	0.00	97.0	0.6
**Diagnostic test done- Blood test**	Gold standard coverage	43.3(4,89)	988.7	4	0.00	99.6	1.3
	Survey reported coverage	53.5(17.1,87.9)	514.4	4	0.00	99.2	0.8
	Don't Know response in survey	17.1(3.5,37.6)	178.0	4	0.00	97.8	0.3
	Agreement	83.9(60.2,98.2)	233.6	4	0.00	98.3	0.4
	Sensitivity	8.2(0,29.2)	23.4	4	0.00	82.9	0.2
	Specificity	97.7(93,100)	27.8	4	0.00	85.6	0.0
	Positive Predictive Value	45.8(6.9,87.8)	15.8	2	0.00	87.3	0.5
	Negative Predictive Value	83.5(62.1,97.2)	179.9	4	0.00	97.8	0.3
**Feeding support- NG feeding**	Gold standard coverage	2.2(0.3,5.2)	21.7	4	0.00	81.5	0.0
	Survey reported coverage	2.9(1.7,4.4)	4.9	4	0.30	18.3	0.0
	Don't Know response in survey	2.7(0.9,5.2)	11.6	4	0.02	65.6	0.0
	Agreement	97.1(93.9,99.2)	16.8	4	0.00	76.1	0.0
	Sensitivity	44.3(11.7,79.1)	4.7	3	0.20	35.8	0.1
	Specificity	98.3(96.9,99.4)	5.8	4	0.21	31.1	0.0
	Positive Predictive Value	24.6(7.3,45.9)	1.9	3	0.59	0.0	0.0
	Negative Predictive Value	99.2(97,100)	16.3	4	0.00	75.4	0.0
**Feeding support- IV fluid**	Gold standard coverage	59.1(42.8,74.5)	100.6	4	0.00	96.0	0.1
	Survey reported coverage	12.7(3.4,26.4)	99.0	4	0.00	96.0	0.1
	Don't Know response in survey	2.7(0.9,5.2)	11.6	4	0.02	65.6	0.0
	Agreement	49.2(35,63.6)	67.0	4	0.00	94.0	0.1
	Sensitivity	15.5(3.3,33.9)	85.7	4	0.00	95.3	0.2
	Specificity	93(85.5,98.1)	16.1	4	0.00	75.1	0.0
	Positive Predictive Value	84(76,91)	3.0	4	0.56	0.0	0.0
	Negative Predictive Value	44.1(28.6,60.2)	71.0	4	0.00	94.4	0.1
**Feeding support- Cup feeding**	Gold standard coverage	6.4(1.5,14)	58.5	4	0.00	93.2	0.1
	Survey reported coverage	18(7,32.5)	92.3	4	0.00	95.7	0.1
	Don't Know response in survey	2.7(0.9,5.2)	11.6	4	0.02	65.6	0.0

	Agreement	81.4(68.9,91.3)	66.9	4	0.00	94.0	0.1
	Sensitivity	38.4(15.3,64.2)	9.0	3	0.03	66.8	0.2
	Specificity	83.9(69.7,94.3)	84.1	4	0.00	95.2	0.1
	Positive Predictive Value	13.6(0.5,35.1)	23.6	4	0.00	83.1	0.2
	Negative Predictive Value	96(90,99.5)	31.7	4	0.00	87.4	0.1
**Phototherapy given**	Gold standard coverage	26.2(11.1,45)	146.0	4	0.00	97.3	0.2
	Survey reported coverage	21.9(8.3,39.4)	120.7	4	0.00	96.7	0.2
	Don't Know response in survey	3.4(1,7)	18.9	4	0.00	78.9	0.0
	Agreement	84.1(73.8,92.3)	51.1	4	0.00	92.2	0.1
	Sensitivity	59.8(44.1,74.7)	14.7	4	0.01	72.7	0.1
	Specificity	90.7(82.1,96.8)	35.1	4	0.00	88.6	0.1
	Positive Predictive Value	64.8(44.4,82.9)	23.6	4	0.00	83.1	0.2
	Negative Predictive Value	87.5(75.8,95.9)	55.0	4	0.00	92.7	0.1
